# Blood donation criteria in Australia: Population knowledge, misperceptions, and impact on donation intent

**DOI:** 10.1111/trf.18474

**Published:** 2025-11-09

**Authors:** Yasmin Mowat, Veronica Hoad, Bridget Haire, Barbara Masser, Anita Heywood, Rachel Thorpe, Hamish McManus, John Kaldor, Skye McGregor

**Affiliations:** ^1^ The Kirby Institute University of New South Wales Sydney New South Wales Australia; ^2^ Australian Red Cross Lifeblood Melbourne Victoria Australia; ^3^ School of Population Health University of New South Wales Sydney New South Wales Australia; ^4^ School of Psychology The University of Queensland Brisbane Queensland Australia

**Keywords:** blood donation, criteria, deferrals, eligibility, knowledge, misconceptions, misperceptions, perceptions

## Abstract

**Introduction:**

Misperceptions about blood donation eligibility can deter individuals from donating. In Australia, misperceptions of one's eligibility status may be exacerbated by recent changes to donor criteria. However, public knowledge of these criteria remains underexplored.

**Methods:**

This study examines the accuracy of perceived eligibility and knowledge of blood donation criteria using data from a nationally representative survey of the Australian population.

**Results:**

Among the 5178 respondents, knowledge of blood donation criteria was limited, with 29.4% of non‐donors unaware of their existence. Of the 57.3% classified as eligible for blood donation at the time of survey completion, 12.4% incorrectly believed they were ineligible and 13.3% did not know their status. Among those classified as ineligible, 55.8% misjudged their status; 36.6% mistakenly believed they were eligible, while 19.2% were uncertain. Those who believed they were eligible were most likely to intend to donate in the next 6 months (35.1%). Overall, knowledge of deferral periods relating to common exclusion factors was poor.

**Conclusion:**

This study indicates a substantial lack of awareness of blood donation criteria and highlights widespread misperceptions and poor knowledge of the criteria in Australia. While most eligible individuals correctly recognized their eligibility, many others may be unnecessarily self‐deferring due to the mistaken belief that they are ineligible. Blood collection agencies should design tailored communication strategies to ensure that eligible individuals are aware of their eligibility, and those who are ineligible understand their status and are informed about when they might become eligible to donate.

## 1 INTRODUCTION

Without an adequate and safe blood supply, medical interventions that rely on blood products would be compromised, leading to increased morbidity and mortality rates. In Australia, an estimated 57.3% of Australians aged 18–74 years old were eligible to donate blood in 2023, yet only 2.8% of the age‐eligible population did so that year.^1^, ^2^ This highlights a significant gap between eligibility and participation, indicating that barriers beyond eligibility shape donation rates. A recent nationally representative survey^1^ explored public perceptions of blood donation eligibility, and revealed widespread misperceptions that may help explain this discrepancy.

The safety of the blood collection and utilization system relies on criteria designed to safeguard the health of donors and minimize the risk to recipients of transfusion‐transmissible infections. These criteria are not static; they evolve over time in response to emerging scientific evidence and technologies, public health considerations, and societal changes. In Australia, recent changes include revisions to eligibility among men who have sex with men^3^, ^4^, ^5^; people with tattoos^6^; removal of the upper age limit for returning donors (previously 80 years); increasing the upper age limit for new donors from 70 to 75 years^7^; and removal of the UK residency deferral.^8^ Relaxing deferral criteria can significantly expand the pool of individuals eligible to donate.^1^ However, policy change alone does not guarantee an increase in donations. Its impact depends largely on public awareness and understanding. Individuals who remain unaware of their revised eligibility or who continue to self‐defer due to outdated perceptions may not present to donate, limiting the potential benefit of such policy shifts. For example, cessation of the vCJD/UK residency deferral in 2022 resulted in an estimated additional 4.4% of Australians aged 18–74 years (approximately 787,600 people based on 2021 Census data) becoming eligible to donate blood.^1^, ^9^ In the first 6 months of the policy change, 32,358 of these newly eligible individuals successfully donated.^10^ While this represents a positive increase in donor numbers, it remains a small fraction of those who became eligible.

Factors that contribute to non‐participation in blood donation include lack of time, fear of an adverse reaction such as fainting, fear of needles or pain, and the perception of being ineligible to donate.^11^, ^12^ In Australia, a recent nationally representative survey revealed widespread miseperceptions of eligibility.^1^ These misperceptions may lead to non‐donation in two ways: individuals who are eligible to donate blood but believe they are ineligible may self‐defer, while those who are ineligible might present to donate and receive a deferral, discouraging future donation when the reason for deferral no longer applies.^13^, ^14^, ^15^, ^16^, ^17^ Targeted education campaigns and clear public messaging are essential to realize the full benefit of policy changes, and to improve public understanding of common exclusion factors. To gain deeper insight into perceptions of blood donation eligibility we analyzed data from the aforementioned national survey to identify the extent of misperceptions about deferral periods and exclusion criteria and assess the relationship between perceived eligibility and intention to donate.

## 2 METHODS

We conducted a nationally representative cross‐sectional survey via the Life in Australia™ probability‐based panel in November 2021. All respondents were resident in Australia and aged 18 years or over. Survey responses were calibrated to population benchmarks using general regression (GREG) calibration estimation.^18^ Further details on data collection, weighting, and ethical approval have been described previously.^1^, ^19^, ^20^ All proportions reported in the results are weighted, with 95% confidence intervals.

A total of 5178 respondents completed the survey, most online (96.5%), with a small proportion via phone (3.5%). Characteristics of the panel have been described previously.^1^, ^19^, ^20^

Respondents were considered eligible to donate if they met guidelines for any blood product (i.e., whole blood, plasma, or platelets). Based on their responses to survey questions, each survey participant was categorized as either eligible to donate, temporarily ineligible, or permanently ineligible according to criteria current at the time of survey (i.e., November 2021, before the UK residency/vCJD risk exclusion was removed), and at the time of analysis (May 2025). Further detail on the assessment of eligibility has been described previously.^1^

Prior to being asked the questions to determine eligibility, respondents were asked “Before starting this survey, did you know there are eligibility criteria for donating blood in Australia?” Responses to this question were categorized by donor status, defined in accordance with Lifeblood's definitions: Current donors (those who had donated any blood product within the last 2 years), lapsed donors (donated any blood product in Australia but over 2 years prior), and non‐donors (never donated any blood product in Australia). A small number of respondents (*n* = 37, 0.7% of the sample) did not provide sufficient information to determine their donor status. While included in the overall analysis, they are excluded from any results reported by donor status category.

To minimize the possibility that respondents might infer eligibility rules from the survey itself, they were also asked early on whether they believed they were currently eligible to donate blood: “To the best of your knowledge, do you think you are currently eligible to donate blood (blood, plasma, or platelets) in Australia?” The accuracy of respondents' perceived eligibility status and their knowledge of the criteria were assessed based on guidelines in effect at the time of data collection. Intention to donate blood within the next 6 months was categorized by respondents' perceptions of eligibility.

Respondents were further asked “How confident are you that you are eligible (or not eligible, depending on their self‐perception of eligibility) to donate blood?” Confidence was measured on a 5‐point Likert scale ranging from 1 (not at all confident) to 5 (extremely confident). Responses were subsequently categorized based on the accuracy of respondents' self‐perceived eligibility.

The survey included questions to assess respondents' knowledge of blood donation criteria, focusing on several of the most common exclusion factors whose population prevalence have been previously estimated.^1^ For each item assessing knowledge of eligibility, accuracy was determined based on eligibility for any type of blood donation (whole blood, plasma, platelets), with the least restrictive criterion considered correct. For example, if an exclusion factor makes an individual ineligible to donate whole blood but still eligible to donate plasma, a response based on the plasma eligibility timeframe was considered correct. Guidance on this process was conveyed to respondents in the survey. One question focused specifically on respondents' knowledge of the criteria related to aspirin, a commonly used medication that does not result in deferral from blood donation.

## 3 RESULTS

When applying the results to deferral criteria at the time of data collection (i.e., November 2021, prior to removal of the UK residency/vCJD risk exclusion), 52.9% of respondents aged 18–74 years old were eligible to donate any blood product. Of those eligible, 14.2% were current donors, 29.4% were lapsed donors, and more than half (55.8%) were non‐donors. At the time of analysis (May 2025), 57.3% of the survey sample aged 18–74 years were eligible. The remaining 42.7% were either temporarily (25.3%) or permanently ineligible (17.4%). Eligibility was higher among men (62.6%) than women (52.8%). The most common reason for exclusion was a diagnosis of anemia or iron deficiency within the last 6 months (9.9%), a history more common among women (16.4%) than men (2.1%). Exclusion rates for specific factors have been described in more detail previously.^1^

Rates of responses to the question on knowledge of eligibility criteria for blood donation in Australia, categorized by donor status, are displayed in Table 1. Those who responded in the affirmative comprised 78.6% (95%CI 76.9%–80.1%) of the sample, while 17.6% (95%CI 16.1%–19.2%) said they were not aware that there are eligibility criteria for blood donation, and 3.8% (95%CI 3.1%–4.6%) said they do not know. Awareness of blood donation criteria varied by donor status. Among current donors, 98.8% knew that eligibility criteria for blood donation exist, compared to 88.1% of lapsed donors and 70.6% of non‐donors.

**TABLE 1:** Awareness of eligibility criteria by donor status.

Before starting this survey, did you know there are eligibility criteria for donating blood in Australia?	Donor status			
	Current donor (*n* = 469, 9.1%)	Lapsed donor (*n* = 1861, 35.9%)	Non‐donor (*n* = 2811, 54.3%)	Total
	% (95%CI)			
Yes	98.8 (97.5–99.5)	88.1 (85.8–90.1)	70.6 (68.1–73.0)	78.6 (76.9–80.1)
No	1.2 (0.5–2.5)	7.6 (6.2–9.2)	25.4 (23.1–27.9)	17.6 (16.1–19.2)
Don't know	0.0	4.3 (2.9–6.3)	3.9 (3.1–5.0)	3.8 (3.1–4.6)
Refused to answer	0.0	0.0	0.0	0.1 (0.0–0.3)

As shown in Table 2, most of those assessed as eligible at the time of survey completion correctly perceived themselves as so (74.2%, 95% CI 71.7%–76.7%). However, 12.5% (95% CI 10.7%–14.5%) believed they were ineligible and 13.3% (95% CI 11.4%–15.4%) did not know whether they were eligible. Of those classified by the survey responses as ineligible, 46.2% (95% CI 43.6–48.7%) correctly identified their status, 34.0% (95% CI 31.6%–36.6%) incorrectly believed they were eligible and 19.8% (95% CI 17.7%–22.1%) did not know.

**TABLE 2:** Perceived eligibility compared to actual eligibility.

Eligibility based on assessment	Perception of eligibility %, 95%CI (% of total Australian population aged over 18 years old)			
	Eligible	Ineligible	Don't know	Total
Eligible	74.2, 71.7–76.7 (36.5%)	12.5, 10.7–14.5 (6.2)	13.3, 11.4–15.4 (6.5)	100 (49.2%)
Ineligible	34.0, 31.6–36.6 (17.3)	46.2, 43.6–48.7 (23.5)	19.8, 17.7–22.1 (10.1)	100 (50.8%)

As shown in Table 3, most respondents reported feeling confident in their self‐perception of eligibility to donate blood. This level of confidence was slightly higher in those whose perception was correct, with 28.5% (95% CI 26.1%–31.0%) “somewhat” confident, 22.9% (95% CI 20.7%–25.3%) “very” confident, and 38.3% (95% CI 35.8%–40.9%) “extremely” confident, as compared to those who were incorrect (35.7%, 25.4%, and 23.1% respectively). As presented in Table 4, those who believed they were eligible were more likely to intend to donate in the next 6 months (35.1%) than those who believed they were ineligible (9.5%), or who were unsure of their eligibility (7.2%).

**TABLE 3:** Confidence in perceptions of eligibility to donate blood.

How confident are you that you are eligible/ineligible to donate blood?	Correctly believes eligible	Correctly believes ineligible	Incorrect: Eligible, but thinks ineligible	Incorrect: Ineligible but thinks eligible
	% (95% CI)			
Not at all confident	0.6 (0.3–1.2)	3.5 (2.3–5.4)	3.4 (1.7–6.8)	1.8 (1.1–2.8)
Slightly confident	7.8 (5.8–10.4)	5.9 (4.0–8.5)	12.2 (6.9–20.6)	11.3 (8.7–14.4)
Somewhat confident	26.6 (23.6–29.9)	21.3 (18.4–24.5)	30.8 (24.4–38.1)	33.5 (29.2–38.1)
Very confident	34.3 (31.1–37.5)	31.2 (28.2–34.3)	27.3 (21.1–34.5)	32.4 (28.1–37.0)
Extremely confident	30.5 (27.6–33.6)	38.0 (34.6–41.6)	25.8 (18.8–34.3)	20.3 (16.8–24.3)
Don't know	0.1 (0.0–0.6)	0.1 (0.0–0.5)	0.5 (0.1–1.8)	0.8 (0.4–1.7)
Refused to answer	0.7 (0.0–0.5)	0.0	0.0	0.0

**TABLE 4:** Intention to donate blood within the next 6 months, by respondents' perceptions of eligibility.

	Do you believe you are eligible to donate blood in Australia?			
	(%, 95% CI)			
	Yes	No	Don't know	Refused to answer
Do you intend to donate blood in the next 6 months?				
Yes	35.1 (32.5–37.8)	9.5 (7.6–11.6)	7.2 (5.4–9.5)	0.0
No	37.5 (34.8–40.1)	72.8 (69.5–75.9)	41.5 (37.0–46.2)	71.4 (30.0–93.6)
Don't know	26.9 (24.5–29.5)	16.6 (14.0–19.6)	49.3 (44.4–54.2)	10.1 (1.8–40.6)
Refused to answer	0.5 (0.3–0.9)	1.1 (0.5–2.4)	2.1 (0.7–5.6)	18.5 (3.0–62.2)

Respondents' knowledge of deferral periods and certain exclusion factors was assessed (Tables 5 and 6). Most respondents correctly identified that HIV (85.8%), hepatitis C (77.5%), and blood cancers (80.4%) lead to permanent deferral, but 8.3%, 9.8%, and 9.2% incorrectly believed HIV, hepatitis C and blood cancers respectively do not result in exclusion. Just over half of respondents (54.1%) correctly recognized that non‐blood cancers do not always result in permanent ineligibility, yet 16.8% incorrectly believed that they do result in permanent ineligibility. Only 10.9% correctly identified that hepatitis B does not lead to a permanent deferral, though eligibility depends on having sufficient protective antibodies (anti‐HBs ≥100 IU/L). A substantial proportion of respondents answered “don't know,” to these questions about health conditions and eligibility, particularly for non‐blood cancers (28.8%) and mental health conditions (20.3%). As shown in Table 6, less than 55% of the sample selected the correct response (true or false) for each of a series of statements about specific health conditions, and for several questions the proportion correct was much lower.

**TABLE 5:** Knowledge of health conditions and deferral periods.

Do the following conditions make someone ineligible to donate blood for the rest of their life….	Prevalence of exclusion factor among 18‐74‐year‐olds^1^	Yes	No	Don't know	Refused to answer
	% (95% CI)				
Mental health conditions	15.4% (13.9–17.0)^a^	12.8 (11.5–14.2)	**66.5 (64.6–68.3)**	20.3 (18.8–21.9)	0.4 (0.2–0.8)
Hepatitis C	1.0 (0.7–1.4)	**77.5 (75.7–79.1)**	9.8 (8.7–11.1)	12.3 (11.0–13.6)	0.5 (0.2–1.2)
Hepatitis B^b^	1.3 (0.9–1.8)	75.2 (73.5–76.9)	**10.9 (9.7–12.1)**	13.4 (12.2–14.8)	0.5 (0.2–1.2)
HIV	0.2 (0.1–0.4)	**85.8 (84.4–87.2)**	8.3 (7.3–9.4)	5.4 (4.6–6.4)	0.5 (0.2–1.2)
Blood cancers	0.5 (0.3–0.8)^c^	**80.4 (78.8–81.8)**	9.2 (8.1–10.3)	10.1 (9.0–11.3)	0.4 (0.2–0.8)
Non‐blood cancers	1.0 (0.7–1.4)^d^	54.1 (52.2–56.0)	**16.8 (15.5–18.2)**	28.8 (27.0–30.6)	0.3 (0.1–0.7)

*Note*: Bold values indicate correct responses.

^a^ Reported having been diagnosed with a mental health condition (mild to severe).

^b^ Eligible if past infection and anti‐HBs ≥100 IU/L.

^c^ Reported having been diagnosed with a blood cancer in the previous 5 years.

^d^ Reported having been diagnosed with a cancer other than blood cancer in the past 5 years.

**TABLE 6:** True or false statements regarding health conditions and criteria for blood donation.

A person who…	True	False	Don't know	Refused to answer
	% (95% CI)			
Has high blood pressure is not eligible to donate at all	25.3 (23.6–27.1)	**38.0 (36.1–40.0)**	36.6 (34.7–38.5)	0.1 (0.1–0.4)
Has anemia or low iron stores cannot donate for a minimum of 6 months^a^	**54.1 (52.1–56.0)**	12.2 (10.9–13.6)	33.5 (31.7–35.4)	0.2 (0.1–0.4)
Has osteoarthritis or osteoporosis is not eligible to donate	18.9 (17.3–20.5)	**39.4 (37.6–41.3)**	41.4 (39.4–43.3)	0.4 (0.1–1.0)
Has asthma or chronic airways disease is not eligible to donate	18.2 (16.7–20.0)	**47.8 (45.8–49.7)**	33.9 (32.1–35.7)	0.2 (0.1–0.5)
Has had a previous stroke is not eligible to donate	**34.4 (32.5–36.3)**	24.4 (22.8–26.1)	41.1 (39.1–43.0)	0.2 (0.1–0.5)
Has diabetes is not eligible to donate	32.8 (31.0–34.7)	**30.2 (28.4–31.9)**	36.8 (34.9–38.7)	0.3 (0.1–0.6)

*Note*: Bold values indicate correct responses.

^a^ Otherwise healthy blood donors who are diagnosed with iron deficiency on blood donation screening are now eligible to donate via apheresis, but they were ineligible at the time of the survey.

**FIGURE 1:**
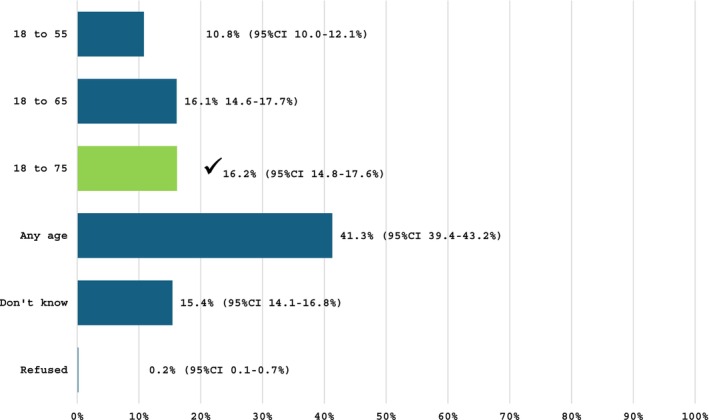
To be eligible to donate blood as a new donor, a person must be aged. ✓ indicates correct responses.

**FIGURE 2:**
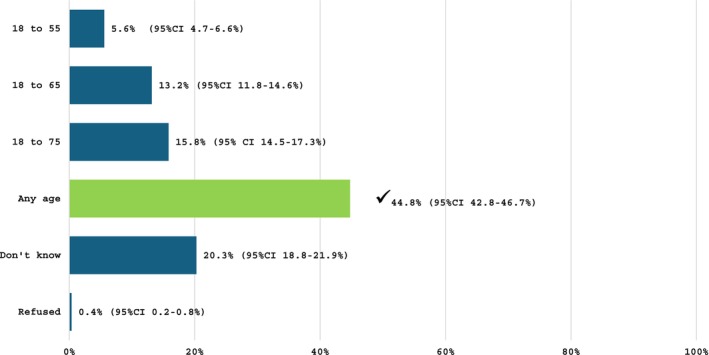
If someone is a current donor, how old do they have to be to be eligible to donate blood again? ✓ indicates correct responses.

As shown in Figure 1, 16.2% correctly answered that a new donor must be aged 18–75 years old in Australia. The rate of correct responses regarding the age requirement of a current donor (any age above 18 years old) was substantially higher, at 44.8% (Figure 2). Under one third (29.3%) of the sample correctly answered that a donor must weigh at least 50 kg to be eligible to donate blood in Australia (see supplemental material, Figure S1), while 10.2% correctly indicated that a donor who has had a tattoo in Australia at a commercial and/or licenced venue can donate with no deferral, a relatively recent policy shift (see supplemental material Figure S2).^6^

Of those taking aspirin (12.1% of respondents, *n* = 627), 40.4% (95% CI 33.5%–47.8%) answered correctly that this does not make one ineligible to donate blood. A quarter (25.9%, 95%CI 20.4%–32.4%) indicated that it did, and one third (33.6%, 95%CI 27.4%–40.4%) said they do not know.

## 4 DISCUSSION

This research indicates a substantial lack of awareness and knowledge of blood donation criteria among the general population in Australia. While most respondents were aware that there are eligibility criteria to donate blood, many respondents (approximately 21%) indicated that they were unaware such criteria existed before they competed the survey, including almost one third of non‐donors (29.4%). Furthermore, a substantial proportion of respondents incorrectly perceived their status with regards to eligibility for blood donation, with 12.5% and 13.3% of the 52.9% who were eligible believing they are ineligible or not knowing they are eligible respectively, and 34.0% and 19.8% of those who were ineligible believing they are eligible or not knowing they are ineligible respectively. Many people may be self‐deferring, or disregarding blood donation as a possibility because they perceive themselves ineligible when they are, in fact, eligible. Respondents who believed they were eligible to donate blood were far more likely to intend to donate in the next 6 months compared to those who believed they were ineligible, highlighting the necessity for targeted educational interventions to improve the public's perception of eligibility.

The high level of confidence reported by respondents regarding their perceived eligibility, regardless of whether their perception was accurate, presents a significant challenge for public education. Confidence in one's beliefs can make one resistant to change, as individuals may not feel the need to seek further information or reconsider their assumptions.^21^ This underscores the importance of tailored education campaigns that not only provide accurate information but also actively challenge misconceptions in a way that resonates with diverse donor groups.

The responses to questions on deferral timeframes for blood donation indicate that knowledge of the criteria among the general population is poor, and several of these criteria relate to factors that are prevalent in the population. For example, most respondents were incorrect or did not know the correct age criteria that currently applies to new blood donors. Many respondents underestimated the age limit, with 10.8% and 16.1% believing it to be 18–55 and 18–65 years old, respectively. Furthermore, less than half (44.8%) of respondents knew that to be eligible to donate blood as a current donor, they can be any age. This indicates that there may be people aged 55 years and older who are self‐deferring. Evidence suggests that donors aged 50+ have higher return rates,^22^, ^23^ and generally experience fewer adverse events than younger donors.^24^ As this age group forms a large proportion of the Australian population (36%),^9^ and tends to donate blood more frequently,^2^ improving public understanding of age‐related eligibility could be a valuable focus for education campaigns aimed at boosting donation rates.^25^

When determining which misperceptions of the criteria to target, blood collection agencies should consider the prevalence of the exclusion factor within the general population, the potential impact of the misperception on product safety, and the length of the deferral. For example, while three quarters (75.2%) of respondents misunderstood, or were unaware (13.4%) of the criteria for people who have/have had hepatitis B, the prevalence of hepatitis B within the general population is relatively low.^26^ Correcting this misperception may only lead to a small increase in potential donors. In contrast, conditions like chronic respiratory diseases, osteoarthritis, and mental health issues are more prevalent in the general population.^27^, ^28^, ^29^ Although fewer respondents misunderstood these eligibility criteria, correcting them may have a greater impact. In addition to correcting misperceptions about common conditions, it would be effective to correct misunderstandings about commonly taken medications. For example, more than half of the 12% of survey respondents who reported taking aspirin incorrectly believed it made them ineligible to donate blood or were unsure of their eligibility.

The need for education on exclusion factors and deferral timeframes is ongoing, and especially important when changes are made to the criteria, to ensure that knowledge of the criteria is current. Given the importance of the continuous evaluation of exclusion factors, as demonstrated by the removal of the vCJD restriction,^10^ it is imperative for blood collection agencies to prioritize continuous refinement of the criteria and effective public health campaigns. With further changes pending, such as changes to deferrals applied due to sexual risk factors which may impact significantly on blood donation eligibility,^4^, ^5^ effective education campaigns are paramount to inform newly eligible donors. Such education campaigns may include social media initatives, targeted email or SMS messages to registered donors, university and workplace engagement, and public awareness efforts through traditional media such as radio, TV, and print.

While the survey did explicitly state that the questions were relating to whole blood, plasma and/or platelets, there may be confusion over the type of donation the criteria were referring to, and responses may have been influenced by the different criteria for different donations. For example, in the question on tattoos, “donating blood” was defined in the survey as encompassing whole‐blood, plasma, and platelets. As there is no deferral period for donation following a tattoo for plasma, the correct answer is “as soon as they want to.” Only 10.2% answered this question correctly. However, the option of 4 months may have been chosen by those who thought only of whole blood in responding, which was correct at the time for whole blood donation (the criterion has since changed and one can now donate whole blood 1 week after having a tattoo in Australia).^6^ Some individuals may be unaware that deferral from whole blood donation does not always preclude eligibility for other donation types, which again calls for improved education, as people may be self‐deferring for longer than necessary.

Comparative research on knowledge of blood donation eligibility among the general population is scarce. Most existing studies focus on healthcare workers or students in lower‐ and middle‐income countries. The only general population study available, conducted in Belgium, assessed knowledge of the deferral criteria for men who have sex with men and similarly found low levels of awareness.^30^ Our study builds on this by examining multiple criteria and deferral timeframes among Australians. These consistent findings suggest that gaps in understanding eligibility may be common internationally, and underscore the importance of tailored educational interventions to improve donor recruitment and retention. Continued research in this area should remain a priority to inform the design and evaluation of education strategies.

In conclusion, this study highlights a critical gap in awareness and understanding of blood donation criteria among the Australian general population. Targeted educational interventions are required to improve public awareness and correct misconceptions, particularly regarding deferral timeframes and criteria for different types of blood donations. Addressing prevalent misperceptions, especially those related to age and common health conditions, such as mental health conditions and asthma, common medications and other factors such as tattoos, may significantly enhance the recruitment and retention of eligible donors. As the landscape of blood donation criteria evolves, ongoing education and reassessment of exclusion factors will remain crucial to ensure that potential donors are well‐informed and able to contribute to the vital supply of blood products.

## FUNDING INFORMATION

This work was majority funded under a Partnership Project grant from the Australian National Health and Medical Research Council (NHMRC APP1151959). Australian governments fund Australian Red Cross Lifeblood to provide blood, blood products and services to the Australian community.

## CONFLICT OF INTEREST STATEMENT

Investigators Hoad and Thorpe are employed by Lifeblood, and investigator Masser's position at the University of Queensland is co‐funded by Lifeblood. Investigator Mowat derives income from the NHMRC Partnership Project grant. The authors declare they have no other conflicts of interest relevant to the manuscript submitted to TRANSFUSION.

## Supporting information


Supplementary Figure S1.

Supplementary Figure S2.

## Data Availability

The data that support the findings of this study are available from the corresponding author upon reasonable request.
