# Supplementary material for: Blood donation criteria in Australia: Population knowledge, misperceptions, and impact on donation intent
Source: Transfusion. 2025 Nov 9;65(12):2336–44. doi: 10.1111/trf.18474 (PMC12704694; doi:10.1111/trf.18474)
Supplement: Supplementary file 1 — Supplementary Figure S1. [file TRF-65-2336-s002.docx]

✓ Indicates correct responses.
